# Resolution of tonic concentrations of highly similar neurotransmitters using voltammetry and deep learning

**DOI:** 10.1038/s41380-024-02537-1

**Published:** 2024-04-25

**Authors:** Abhinav Goyal, Jason Yuen, Stephen Sinicrope, Bailey Winter, Lindsey Randall, Aaron E. Rusheen, Charles D. Blaha, Kevin E. Bennet, Kendall H. Lee, Hojin Shin, Yoonbae Oh

**Affiliations:** 1 Medical Scientist Training Program, Mayo Clinic, Rochester, MN 55905 USA; 2 Department of Neurologic Surgery, Mayo Clinic, Rochester, MN 55905 USA; 3 Department of Neurosurgery, Southmead Hospital, Bristol, BS10 5NB UK; 4 Department of Neuroscience, University of Chicago, Chicago, IL 60637 USA; 5 Department of Neurosurgery, Johns Hopkins University, Baltimore, MD 21287 USA; 6 Division of Engineering, Mayo Clinic, Rochester, MN 55905 USA; 7 Department of Biomedical Engineering, Mayo Clinic, Rochester, MN 55905 USA

**Keywords:** Neuroscience, Molecular biology

## Abstract

With advances in our understanding regarding the neurochemical underpinnings of neurological and psychiatric diseases, there is an increased demand for advanced computational methods for neurochemical analysis. Despite having a variety of techniques for measuring tonic extracellular concentrations of neurotransmitters, including voltammetry, enzyme-based sensors, amperometry, and in vivo microdialysis, there is currently no means to resolve concentrations of structurally similar neurotransmitters from mixtures in the in vivo environment with high spatiotemporal resolution and limited tissue damage. Since a variety of research and clinical investigations involve brain regions containing electrochemically similar monoamines, such as dopamine and norepinephrine, developing a model to resolve the respective contributions of these neurotransmitters is of vital importance. Here we have developed a deep learning network, DiscrimNet, a convolutional autoencoder capable of accurately predicting individual tonic concentrations of dopamine, norepinephrine, and serotonin from both in vitro mixtures and the in vivo environment in anesthetized rats, measured using voltammetry. The architecture of DiscrimNet is described, and its ability to accurately predict in vitro and unseen in vivo concentrations is shown to vastly outperform a variety of shallow learning algorithms previously used for neurotransmitter discrimination. DiscrimNet is shown to generalize well to data captured from electrodes unseen during model training, eliminating the need to retrain the model for each new electrode. DiscrimNet is also shown to accurately predict the expected changes in dopamine and serotonin after cocaine and oxycodone administration in anesthetized rats in vivo. DiscrimNet therefore offers an exciting new method for real-time resolution of in vivo voltammetric signals into component neurotransmitters.

## Introduction

The modern landscape of neuroelectrochemistry is evolving towards the development of methods which are directly translatable to human research. It is becoming increasingly well understood that many neuropsychiatric disorders, including Parkinson Disease, Huntington Disease, Tourette Syndrome, Alzheimer Disease, schizophrenia, addiction, depression, and chronic pain, have an etiology stemming from an imbalance or disruption in neurochemical signaling. In particular, recent work has shown that it is specifically a disruption in tonic concentrations of neurotransmitters that give rise to the homeostatic imbalances that allow these diseases to develop [1–7]. Briefly, neurotransmitter signaling can be divided into phasic and tonic release. The former refers to burst-firing neurons releasing neurochemicals in response to salient stimuli, and is characterized by short-lived, rapid release of neurochemicals into the synaptic cleft. This type of release can be measured using techniques such as fast-scan cyclic voltammetry (FSCV) [8, 9]. In contrast, tonic-level release of neurotransmitters involves pacemaker-like spontaneous firing of neurons, periodically releasing neurotransmitters into the extrasynaptic space. This extracellular tonic concentration is thought to maintain network homeostasis by modulating neural excitability in response to external stimuli [10–12]. A significant, long-term disruption in this homeostasis would therefore give rise to issues pertaining to over- or under-excitable networks, including aberrant oscillations, higher firing thresholds, changes in receptor densities, and even neural atrophy.

It is therefore of vital importance to continue to develop and refine electrochemical methods for probing the tonic concentrations of neurotransmitters in vivo for future human translation. Methods for measuring these tonic concentrations have been outlined in depth in a recent review [2]. For human translation, one of the most promising branches of these methods is cyclic voltammetry [13–16]. This is because cyclic voltammetry enables measurement in situ without the need for repetitive sample extraction from the brain for laboratory analysis and does not involve genetic modification or viral transduction (cf. methods such as optogenetics). Voltammetry’s relative safety profile, involving the use of a minimally traumatic measurement electrode, makes it the most likely branch of methods for approval in human use in the near future. Voltammetric methods for tonic-level measurements, including multiple cyclic square-wave voltammetry (M-CSWV), N-shaped multiple cyclic square-wave voltammetry (N-MCSWV), and fast scan controlled-adsorption cyclic voltammetry (FSCAV) have shown promise for a variety of applications in animal research, including for probing the neurochemical dynamics of addiction and Tourette Syndrome treatment [2, 17–21].

Since the principle of voltammetry is to identify molecules by the specific voltages at which they are oxidized or reduced, an as-of-yet unsolved problem is the inability to discriminate between structurally similar monoamines, which oxidize or reduce at nearly identical voltages. This is especially a problem in the in vivo environment, where mixtures of similar appearing neurochemicals, such as dopamine and norepinephrine, are abundant. Previous work has attempted to leverage voltammetric measurements in combination with artificial intelligence algorithms, such as principal components regression (PCR) [22], partial least squares linear regression (PLSR) [23], support vector regression (SVR) [24], and deep learning networks [25, 26] to resolve the individual monoamine concentrations present within a mixture. However, these models have currently only been applied to *phasic* neurotransmitter release data and have shown limited success when extended to predict on mixtures in the in vivo environment. Given the importance of tonic concentrations of neurotransmitters, and the recent emergence of voltammetric methods to study them, models for resolving these tonic concentrations of similar appearing monoamines are ready for development.

Here, we introduce DiscrimNet, a deep learning algorithm that can successfully resolve tonic concentrations of neurotransmitters from in vitro mixtures containing both single monoamines and mixtures of dopamine (DA), norepinephrine (NE), and serotonin (5-HT). Further, in the in vivo environment in anesthetized rats, DiscrimNet successfully predicts expected changes in these neurotransmitters after pharmacological intervention with drug of abuse administration. We detail the architecture of DiscrimNet and compare its performance to a variety of shallow learning algorithms that have been used in prior literature to attempt to solve a similar problem. We show that DiscrimNet vastly outperforms all other shallow learning algorithms tested.

## Materials and methods

### Electrode fabrication

Carbon fiber microelectrodes (CFMs) were fabricated using an established standardized CFM design at Mayo Clinic [16]. A single carbon fiber (AS4, diameter = 7 μm; Hexel, Dublin, CA) was inserted into a silica tube (ID = 20 μm, OD = 90 μm, 10 μm coat with polyimide; Polymicro Technologies, Phoenix, AZ). The connection between the carbon fiber and the silica tubing was sealed with epoxy resin. The silica tubing was then attached to a nitinol (Nitinol #1, an alloy of nickel and titanium; Fort Wayne Metals, IN) extension wire with a silver-based conductive paste. The nitinol wire was then insulated with polyimide tubing (ID = 0.0089″, OD = 0.0134, WT = 0.00225; Vention Medical, Salem, NH) up to the exposed carbon fiber tip. The exposed carbon fiber was then trimmed under a dissecting microscope to a length of 50 μm. An Ag/AgCl reference electrode was prepared from Teflon-coated silver wire (A-M systems, Inc., Sequim, WA) by chlorinating the stripped tip in saline with a 9 V dry cell battery. CFMs were chemically tested in a beaker with TRIS buffer prior to coating with a PEDOT:Nafion deposition solution, which has been shown to minimize the effect of in vivo biofouling and increase sensitivity to electroactive monoamine neurotransmitters [27].

### In vitro experiments

**Fig. 1:**
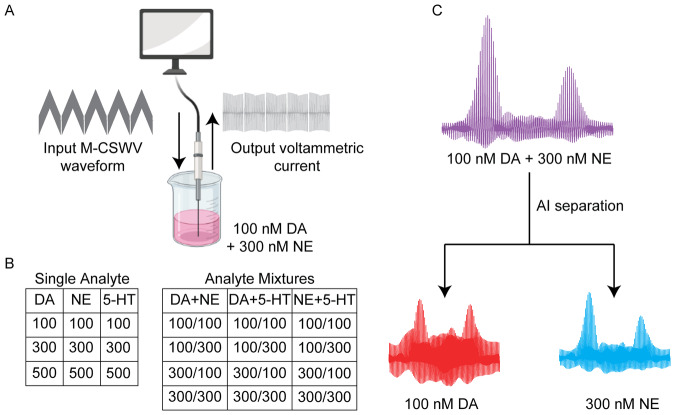
In vitro workflow. **A** The input M-CSWV waveform is transmitted to the CFM, oxidizing and reducing surrounding electrochemical species in the beaker solution. The resulting current data is picked up by the CFM and sent back to the computer for analysis. **B** Tested single and mixture monoamine solution concentrations (nM). **C** Artificial intelligence models are used to resolve mixtures of monoamines into their individual components.

Figure 1 outlines the workflow of the in vitro experiments. M-CSWV was performed using a commercial electronic interface (NI USB-6363, National Instruments) with a base-station desktop computer and software written in-house using MATLAB (MathWorks Inc., Natick, MA) [28]. Data, in the form of a sequence of unsigned 2-byte integers, were saved to the base-station computer. Additional processing, including temporal averaging, filtering, and background current simulation were done in MATLAB [28]. A CFM and Ag/AgCl reference electrode were lowered into the monoamine solution. The NI system transmitted the M-CSWV waveform through the CFM, oxidizing and subsequently reducing surrounding species. The resulting current traveled back through the CFM to the base-station computer for analysis (Fig. 1A).

The monoamine solutions used in this work are shown in Fig. 1B. These three monoamines were chosen because they are commonly seen in the in vivo environment and are relevant for a variety of neuropsychiatric conditions [29–31]. Other potential interferents such as ascorbic acid, DOPAC, and pH changes have already been shown to be excluded by the M-CSWV waveform [32], so were not assessed. All solutions were dissolved in TRIS buffer to the appropriate concentrations. Each solution represents a single dataset per CFM. For each dataset, the CFM was allowed to stabilize in TRIS buffer for 30 minutes. Then, the CFM was inserted into the solution, and was allowed to stabilize for an additional 10 minutes. 50 voltammograms were then recorded (scan repetition rate = 0.1 Hz [32]). In between solutions, the CFM was rinsed with TRIS buffer. A total of 12 CFMs with data collected by 5 different experimenters were used for in vitro data collection.

Voltammogram processing proceeded as described previously [28, 32, 33]. The current data measured by the CFM is sent to the base-station computer for processing. Tonic concentrations are obtained through dynamic background subtraction of the non-Faradaic current. The final background subtracted voltammogram was fed into the artificial intelligence models for neurochemical concentration prediction (Fig. 1C).

### Unlabeled in vivo data

The second branch of our model learns salient features from unlabeled in vivo data. This data was collated from prior in vivo experiments performed in our laboratory. These experiments were all performed on male urethane-anesthetized Sprague-Dawley rats (150–200 g). For each experiment, using M-CSWV, 50 voltammograms were selected randomly from a portion of the experiment after the CFM was electrochemically stabilized but before any experimental manipulations, such as electrical stimulation or drug of abuse administration, were performed. The voltammograms were preprocessed in the same way as the in vitro data. Overall, 20 experiments conducted by 3 different investigators were used, for a total of 500 in vivo voltammograms. The brain regions targeted by these experiments include the nucleus accumbens (*n* = 13), the dorsal hippocampus CA1 region (*n* = 3), and the medial prefrontal cortex (*n* = 4). Of note, the electrodes used within these in vivo experiments are distinct from those used to collect the in vitro data.

### Shallow learning algorithm modeling

All shallow learning modeling was performed in MATLAB. The following artificial intelligence algorithms were assessed based on their previous use in the electrochemical literature for discriminating similar appearing monoamines: Support vector regression (SVR), principal components regression (PCR), partial least squares linear regression (PLSR), lasso regression, ridge regression, and elastic net regression [14, 22, 23, 34, 35]. The 50 voltammograms and the associated concentration labels from each in vitro experiment were compiled and shuffled. Prior literature utilizing artificial intelligence for concentration resolution from mixtures has used the same electrodes to construct the training and test sets. We follow this approach for our “within electrode” training branch. In general, this decision makes sense, as electrochemical data arising from different electrodes can be quite variable, due to different electrode surface characteristics, lengths, etc. These differences lead to differing voltammograms for solutions containing the same concentrations of monoamines, which can disrupt artificial intelligence model training. However, the decision to use the same electrodes for training and testing also presents several problems. When future data is collected with new electrodes, it will mean the model will have to be retrained with data from these new electrodes. Further, this prevents extension to the in vivo environment, as ground truth labeling of in vivo data is difficult to obtain. Therefore, we also included an “across electrode” training branch, in which validation and testing voltammograms were allowed to come from electrodes entirely unseen during model training. This tests the electrode-wise generalizability of each model. Having a model that performs well across-electrodes would be beneficial, as it would mean that data from future electrodes could simply be fed back into the model without the need for retraining. Such generalizability would bode well for generalizing to the in vivo environment. Indeed, our unlabeled in vivo data was collected with an entirely separate set of electrodes from the in vitro data.

**Fig. 2:**
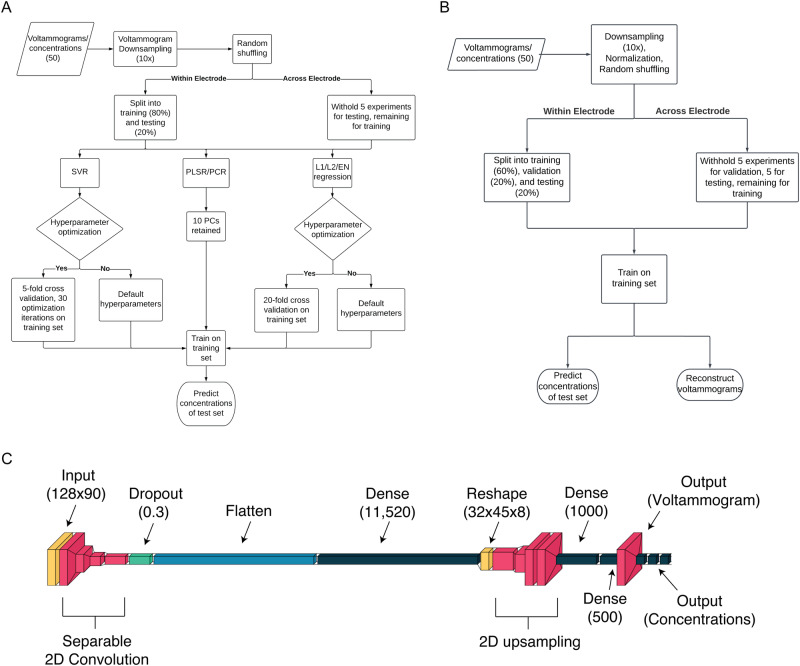
DiscrimNet architecture and artificial intelligence algorithm flowcharts. **A** Shallow learning algorithm evaluation flowchart. The evaluation of each model is handled differently depending on the availability of tunable hyperparameters. All models were evaluated on the same training and test sets. **B** Deep learning algorithm evaluation flowchart. Voltammogram processing proceeds similarly to Fig. 2A. **C** DiscrimNet is a convolutional autoencoder consisting of 2D convolution and MaxPooling blocks in the encoder branch, and 2D convolution and upsampling blocks in the decoder branch.

For the “within electrode” branch, the shuffled voltammogram dataset was split randomly into training and test sets (80% training) for evaluation (Fig. 2A). In total, there were 11350 voltammograms, yielding 9080 voltammograms for training and 2270 for testing. To maximize the generalizability of the models, each model was programmed to output a predicted concentration for each monoamine (DA, NE, 5-HT) regardless of whether the monoamine was present in the mixture. Model performance was evaluated with root mean square error (RMSE) between the predicted and true test concentrations.

For support vector regression, the box constraint, epsilon, and kernel scale hyperparameters were optimized using five-fold cross validation on the training set using the L1QP solver. A total of 30 optimization iterations were performed on the training set before the final concentration predictions on the test set. For PCR and PLSR, a total of 7 principal components were used in the regression, as 7 principal components explained 99% of the variance.

For lasso, ridge, and elastic net regression, Principal Component Analysis (PCA) was performed on the training and test datasets, and the first 100 principal components of the training set were kept for regression. This was to reduce the program execution time and make the regression problem tractable. 20-fold cross validation was used to estimate the coefficients of each regression model. The largest lambda value (retained regularization coefficients) was used such that the cross-validated mean squared error was within 1 standard error of the minimum mean squared error.

### DiscrimNet architecture

M-CSWV-derived voltammograms can be reshaped into 2D heatmaps, which effectively can be fed as images to a deep learning algorithm. Therefore, deep learning network layers which operate on images, such as 2D convolutional layers, can be used on our voltammograms. Such layers offer an advantage over equivalent layers which operate on 1D time series (e.g., 1D convolutional layers, recurrent layers, etc.) because of their ability to encode multi-dimensional spatial information in the data, and to use this encoding to discriminate between similar-appearing voltammograms.

One of the major limitations of in vivo use of voltammetric methods such as M-CSWV is that labeling the concentrations of in vivo voltammograms is difficult, as there is currently no way to accurately determine the concentrations of each neurotransmitter present in a certain brain region. One could use in vivo microdialysis, but this method is unsuitable for future clinical use, due to its propensity for tissue damage, low sampling rate, and need for external laboratory identification [2, 36]. To circumvent these concerns, we have developed a semi-supervised learning algorithm which is trained on labeled in vitro voltammograms, allowed to encode features of unlabeled in vivo voltammograms, and finally can predict concentrations from unseen in vivo data.

DiscrimNet was built as a convolutional autoencoder (Fig. 2C) using the Keras library [37] in Python. Autoencoders, which consist of an encoding (learning) branch and a decoding (reconstruction) branch, are typically used in an unsupervised manner to learn the salient features of the input data and attempt to reconstruct it. Autoencoders have been used for anomaly detection, noise suppression, data augmentation, etc. [38] However, we use it in a semi-supervised manner for both concentration prediction and voltammogram reconstruction.

First, voltammogram pre-processing proceeds by normalizing all pixel values to be between 0 and 1 (as recommended for stable training of deep learning networks) [39]. The autoencoder is trained on the same labeled in vitro data as the shallow learning algorithms were trained on. As before, training proceeded in either a “within electrode” or “across electrode” manner (Fig. 2B).

**Fig. 3:**
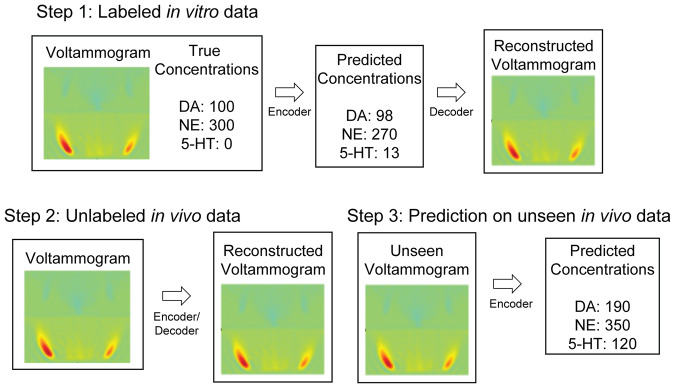
Autoencoder training iterations. First, the full autoencoder is used to both predict concentrations and reconstruct the input voltammogram, while simultaneously minimizing regression and reconstruction loss. Next, both the encoder and decoder weights are transferred to a new autoencoder, which is trained to reconstruct unseen in vivo data. Finally, the fully-trained encoder weights are used to predict concentrations on unseen in vivo data.

The output of the encoding pathway consists of three concentrations, the predictions for DA, NE, and 5-HT, respectively (Fig. 3). The output of the decoding pathway is an attempted reconstruction of the input voltammogram. There are thus 2 losses that the training process attempts to minimize: regression loss, which is the root mean square error between the predicted concentrations and actual concentration, and the reconstruction loss, which is the binary cross-entropy between the input and output voltammograms. By minimizing both, the network is forced to learn the salient features within the voltammogram and allow these features to inform the concentration prediction process.

Second, an identical autoencoder is built, but without the dense blocks that lead to concentration prediction. This autoencoder is only designed for reconstruction. Following the principles of transfer learning, the trained layers from the first autoencoder are transferred to this one, and then this second network is fed unlabeled in vivo voltammograms. Now, the layer weights will be modified to incorporate salient features of in vivo voltammograms.

Finally, an encoder is built, identical to the encoding branch of the first network. The encoding layer weights of the second network, which contains information from labeled in vitro data as well as unlabeled in vivo data, are transferred to this one. Unseen in vivo data can then be fed into this encoder, which will output predicted concentrations for DA, NE, and 5-HT in vivo.

Overall, this convolutional autoencoder derives inspiration from Xue et al., 2021 [40], who utilized a similar autoencoder and transfer learning scheme to predict phasic concentration changes of several monoamines. However, our model possesses some key differences. First, the model of Xue et al. utilized 1D convolution blocks, and reconstructed the voltammogram directly from the concentration predictions. In contrast, our model uses 2D convolution blocks, and reconstructs the voltammogram from a low-dimensional 2D layer, which is near the bottom of the encoding branch. Because our voltammograms possess significantly higher dimensions than theirs (128 × 45,000) vs. (1 × 200), it is impossible to reconstruct the voltammogram from just 3 concentration values. Various additional adjustments are made, such as the addition of batch normalization blocks, the use of Separable convolution blocks rather than simple convolution blocks, and the use of 2D convolution and Upsampling blocks rather than transposed convolution blocks. Finally, the biggest difference is that Xue et al.’s in vitro test set as well as their in vivo set came from the same electrodes used for the training set, while our “across electrode” branch has test sets that consist of voltammograms from electrodes never seen during training. Further, our in vivo dataset comes from an entirely different set of electrodes. Additionally, our model aims to predict tonic concentrations of neurotransmitters, while Xue et al.’s model predicts phasic changes in concentrations.

For all phases of DiscrimNet training, the Adam optimizer was used with random initial hyperparameters. After 3 epochs of no validation loss reduction, learning rate was reduced by 10-fold, with a minimum possible learning rate of 1×10^-6^. Training continued for a maximum of 50 epochs but was programmed to stop after 20 epochs of no reduction in validation loss. For training on labeled in vitro data, the loss functions were root mean square error for the monoamine concentration predictions and binary cross-entropy for voltammogram reconstruction. For training on unlabeled in vivo data, the loss function was binary cross-entropy for voltammogram reconstruction.

### In vitro validation experiments

To validate DiscrimNet’s ability to characterize individual concentrations of DA, NE, and 5-HT, a new set of in vitro solutions were made that contain all three monoamines (Table 1). These mixtures are entirely different from the initial in vitro mixtures used to create the training set, as those mixtures contained either one or two monoamines each. The mixtures here contained three monoamines and would serve purely as a test set (no iteration of the model would be trained on this set of mixtures). A similar procedure was used to construct this test set and pre-process it (Fig. 2). Three CFMs were used to record 20 voltammograms from each of these mixtures. DiscrimNet and SVR were then used to predict the concentrations of DA, NE, and 5-HT of each voltammogram.

**Table 1:** In vitro validation solution concentrations. Each mixture consisted of all three monoamines.

DA (nM)	NE (nM)	5-HT (nM)
100	250	500
300	250	500
100	500	250
300	500	250
500	100	250
500	300	250
500	250	100
500	250	300

### Selectivity validation experiments

Prior work has shown that the voltammograms recorded by M-CSWV are not affected by other common electroactive interferents in the brain, including adenosine, ascorbic acid, DOPAC, pH changes, homovanillic acid (HVA), and uric acid. [32] However, to further confirm that DiscrimNet’s concentration prediction performance would not be affected by these electroactive interferents, we performed two separate in vitro validation experiments across 3 CFMs.

In the first experiment, 20 in vitro voltammograms were collected from solutions that contained 500 nM DA by itself or in the presence of physiologic concentrations of other possible in vivo interferents. Each solution contained 500 nM DA in addition to one of adenosine (1 μM), ascorbic acid (200 μM), HVA (20 μM), DOPAC (20 μM), uric acid (100 μM), or pH change (-0.2). DiscrimNet was then assessed on its ability to predict the correct DA concentration based on the voltammograms. DiscrimNet was not retrained using voltammograms collected with these other interferents.

**Fig. 4:**
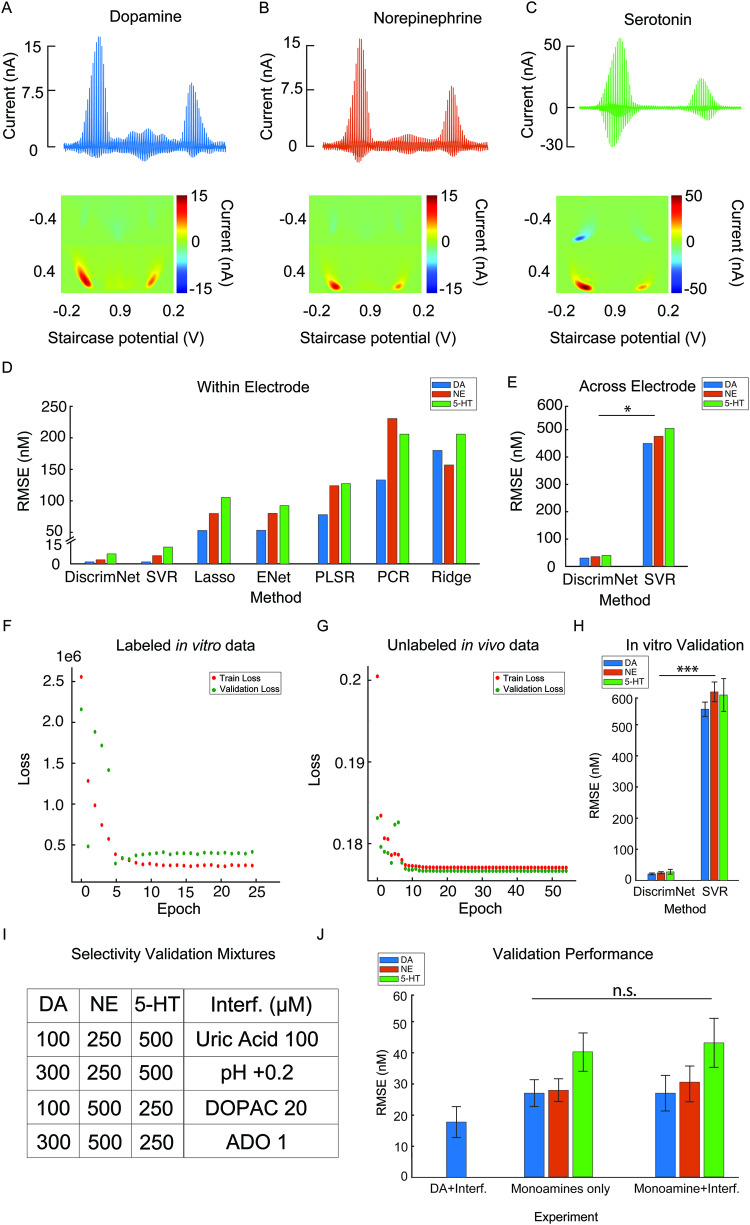
Model output, performance, and validation. **A** 500 nM dopamine. **B** 500 nM norepinephrine. **C** 100 nM serotonin. The voltammograms for DA and NE are very similar due to their structural similarity. **D** Root Mean Square Errors (RMSEs) between actual and predicted monoamine concentrations. The test set consisted of voltammograms obtained using the same electrodes (within) as the training set voltammograms. **E** Same as (**A**), but the test set consisted of voltammograms obtained using different electrodes (across) as those from the training set. **F** Training and validation loss curves for DiscrimNet phase 1, training on labeled in vitro data. **G** Training and validation loss curves for DiscrimNet phase 2, training on unlabeled in vivo data. **H** DiscrimNet and SVR are used to predict concentrations from in vitro mixtures containing all three monoamines. Error bars indicated standard deviation across CFMs. **I** Solutions used for the second selectivity validation experiment. All 4 solutions were measured across 3 CFMs. Monoamine concentrations are in nM. **J** DiscrimNet performance for both selectivity validation experiments, as well as its performance from monoamine mixtures without interferent (same data as (**H**), left). Error bars indicate standard deviation across CFMs.

In the second experiment, 20 in vitro voltammograms were collected from solutions that contained all three analytes of interest (DA, NE, and 5-HT) in various concentrations (Fig. 4I) and in the presence of physiologic concentrations of one other possible in vivo interferent (adenosine, pH change, DOPAC, and uric acid). DiscrimNet was then assessed on its ability to predict the correct analyte concentrations based on the voltammograms. DiscrimNet was not retrained using voltammograms collected with these other interferents.

### In vivo validation experiments

Male Sprague-Dawley rats (*n* = 8; 200 g; Envigo, United States) were used for in vivo validation studies. Rats were kept in social housing in an Association for Assessment and Accreditation of Laboratory Animal Care International (AAALAC) accredited vivarium following a standard 12-h light/dark cycle at constant temperature (21 °C) and humidity (45%) with ad libitum food and water. The present studies were approved by the Institutional Animal Care and Use Committee (IACUC), Mayo Clinic, Rochester, MN. The NIH Guide for the Care and Use of Laboratory Animals guidelines (Department of Health and Human Services, NIH publication No. 86-23, revised 1985) were followed for all aspects of animal care. As animals were not allocated to separate experimental groups, randomization and blinding were not needed.

Rats were anesthetized with urethane (1.5 g/kg, i.p., Sigma-Aldrich, St. Louis, MO, USA). After depth of anesthesia was confirmed with loss of hind limb nociceptive withdrawal response, rats were fixed to a stereotactic surgical frame (David Kopf Instruments, Tujunga, CA, USA). A burr hole was drilled over the right nucleus accumbens core (Coordinates from bregma [41]: AP: +1.2, ML: +1.4, DV: -6.5 to -7.5) for placement of the CFM. Another burr hole was drilled on the contralateral side for placement of the Ag/AgCl reference electrode. The CFM was lowered to the target location, and the M-CSWV signal was allowed to stabilize for 60 minutes. Then, cocaine (2 mg/kg, i.v., *n* = 4) [17] or oxycodone (2.5 mg/kg, i.v., *n* = 4) [42] was administered, and the resulting effects on monoamine concentrations were recorded with M-CSWV.

After the experiment, DiscrimNet was used to predict concentrations for the three monoamines over the course of each in vivo experiment. Each model was evaluated on its ability to track the expected changes in the monoamines in response to cocaine and oxycodone based on their mechanisms of action.

## Results

### In vitro data collection

The 12 CFMs performed similarly in their ability to acquire stable electrochemical recordings across each solution. Representative plots showing background-subtracted voltammograms and the associated color plots for each monoamine demonstrate a high signal-to-noise ratio and high recording fidelity (Fig. 4). The oxidation and reduction voltages derived from the voltammograms for DA and NE are indiscriminable to the naked eye (Fig. 4A, B), confirming the need for artificial-intelligence based post-processing algorithms.

### In vitro model performance

To determine which model performed the best at discriminating between similar-appearing monoamines, we assessed the root mean square error (RMSE) between the models’ concentration predictions and the actual monoamine concentrations for the labeled in vitro data. When the test set consisted of voltammograms held out from the same electrodes used for the training set (“within electrode”), DiscrimNet and support vector regression (SVR) showed significantly lower RMSEs compared to the remaining models, across all monoamines (two-way ANOVA; *p* < 0.001; Fig. 4D). Each model exhibited similar variance across all monoamines (Levene’s Test; *p* > 0.1). This result was likely achieved due to the number of tunable hyperparameters allowed by the SVR architecture, enabling robust fitting to a very high-dimensional dataset. Overfitting to the training set is a natural concern with such a flexible and tunable model but appears to not have occurred here due to the low test errors achieved by SVR, evident in Fig. 4D. Without hyperparameter optimization, SVR showed similar performance to lasso regression (two-way ANOVA, *p* > 0.05), showcasing the utility of hyperparameter tuning.

Because SVR vastly outperformed other shallow learning algorithms in predicting monoamine concentrations, it was the only algorithm to be assessed for the “across electrode” training branch, where test set voltammograms arose from electrodes unseen during model training. In this case, DiscrimNet showed significantly lower RMSEs than SVR across all monoamines on the test set (one-way ANOVA, *p* < 0.001; Fig. 4E). This result showcases the lack of generalizability across electrodes displayed by shallow learning algorithms, as seen in prior literature, which is why test set voltammograms in these prior studies came from the same electrodes as training set voltammograms [22, 23, 40].

The training and validation loss curves for the two training phases of DiscrimNet are shown in Fig. 4F, G. For both training iterations, a plateau is reached early in training, with incremental improvements to validation loss seen after the respective plateaus. This early plateau indicates that DiscrimNet is well suited to the problems of discriminating similar appearing monoamines, as well as reconstructing voltammograms from low dimensional latent space representations. Interestingly, even after training for many epochs following the plateau, overfitting (which would be represented by an increase in validation loss) did not occur to a significant degree. This is possibly due to the presence of dropout and MaxPooling layers within the network, which both serve to eliminate a certain percentage of learned representations during each training epoch. These layers are intended to significantly mitigate overfitting.

Because SVR significantly outperformed the other shallow learning models evaluated, it was the only shallow learning model assessed for in vitro validation experiments. DiscrimNet was shown to significantly outperform SVR in its ability to accurately predict concentrations of DA, NE, and 5-HT from solutions containing all three monoamines (Fig. 4H; one-way ANOVA, *p* < 0.001). This supports DiscrimNet’s generalizability to solutions that differ from the training set (which consisted of solutions containing only one or two of the monoamines) and supports its potential applicability to the in vivo setting.

Finally, to confirm that DiscrimNet’s in vivo performance will not be affected by the presence of other electroactive interferents such as adenosine, uric acid, ascorbic acid, pH changes, DOPAC, or homovanillic acid (HVA), we performed two selectivity validation experiments. In the first, DA concentration was predicted in the presence of one of these interferents. In the second, concentrations from mixtures containing all three analytes of interest (DA, NE, and 5-HT) were predicted in the presence of one of these interferents (Fig. 4I). DiscrimNet’s ability to accurately predict all three analyte concentrations in the presence of interferents was not significantly different from its ability to accurately predict monoamine concentration with no added interferents (one-way ANOVA, *p* > 0.1), as expected from M-CSWV’s ability to exclude these analytes from its voltammograms (Fig. 4J).

### In vivo model performance

**Fig. 5:**
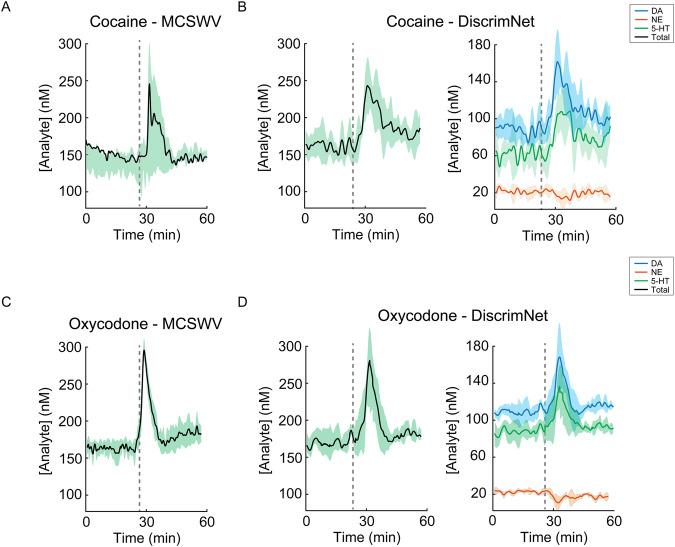
Validation of DiscrimNet on in vivo data. DiscrimNet is used to predict monoamine concentrations from in vivo experiments in which rats were administered cocaine (*n* = 4) and oxycodone (*n* = 4). **A** Total monoamine concentrations averaged across experiments in which cocaine was administered (i.v.) at t = 27 min (dashed gray line) recorded with M-CSWV. **B** DiscrimNet’s prediction of the total monoamine concentrations, and the individual monoamine concentrations. **C** Total monoamine concentrations averaged across experiments in which oxycodone was administered (i.v.) at t = 27 min (dashed gray line) recorded with M-CSWV. **D** DiscrimNet’s prediction of the total monoamine concentrations, and the individual monoamine concentrations. Shaded regions denote standard deviation.

To test DiscrimNet’s ability to predict concentrations of each monoamine in vivo, it was modeled on in vivo data previously acquired in our lab, in which either cocaine or oxycodone was administered to urethane-anesthetized rats, and M-CSWV was used to record the voltammetric signal from the right nucleus accumbens core (Fig. 5). As seen, DiscrimNet accurately predicted the expected DA and 5-HT surges following cocaine and oxycodone administration [17, 18, 42] (Fig. 5A, B). The tonic concentrations predicted by DiscrimNet match those we have previously seen in our lab [17, 42]. Overall, the total monoamine concentration predictions match the trends recorded by M-CSWV, and the individual monoamine concentration predictions follow the outcomes expected from the individual drugs’ mechanisms of action (i.e., increase in both 5-HT and DA, with a larger increase in DA, and no change to NE).

## Discussion

Here we have outlined the development of DiscrimNet, a deep learning network capable of resolving tonic concentrations of DA, NE, and 5-HT from in vitro mixtures and in the anesthetized in vivo environment for the first time with high temporal resolution (~10 s). The architecture of the model is described, and its performance is compared to a variety of shallow learning algorithms, outperforming all in terms of generalizability and in vivo concentration accuracy. After the model is trained, it can be used to predict concentrations of monoamines in real time as the voltammetric signal is obtained.

DiscrimNet vastly outperforms other algorithms in the literature with its generalizability across electrodes and into the in vivo environment in anesthetized rodents. Prior literature utilizing artificial intelligence for concentration resolution from mixtures has used the same electrodes to construct the training and test sets to improve the similarity between training and test voltammograms. However, this decision reduces generalizability of the model to data collected from new electrodes. Therefore, we also included an “across electrode” training branch, in which validation and testing voltammograms were allowed to come from electrodes entirely unseen during model training, and our in vivo data came from electrodes distinct from those used for in vitro data collection and model training. DiscrimNet’s ability to accurately predict concentrations of monoamines from electrodes unseen during training (Fig. 4) is of vital importance, as it allows data collected from new electrodes to be predicted without the need for model retraining and supports DiscrimNet’s application to the in vivo environment.

DiscrimNet’s ability to learn salient features of large quantities of unlabeled in vivo data (Fig. 3, Step 2) lends it a significant advantage compared to shallow learning algorithms which cannot be trained on such data. This advantage is evidenced here, with its ability to accurately track expected changes in neurotransmitters after pharmacological intervention with drugs such as cocaine and oxycodone (Fig. 5). Cocaine is a non-selective reuptake inhibitor, but can be seen here to only elevate DA and 5-HT tonic levels in the nucleus accumbens core, and not NE. This finding is highly consistent with post-mortem analysis of nucleus accumbens core tissue content of these analytes. Post-mortem tissue analysis has confirmed DA as the major catecholamine and 5-HT as the major indoleamine present in the nucleus accumbens core of the mammalian brain with NE comprising less than 1% of the content [43], supporting why we primarily saw increases in only DA and 5-HT following cocaine administration. Further, the absolute concentrations predicted by DiscrimNet match those we have reported previously [17, 18, 32, 42].

Oxycodone is a μ-opioid agonist, and has been shown to bind to μ -opioid receptors on VTA GABAergic interneurons, deactivating these interneurons and disinhibiting dopaminergic transmission to the nucleus accumbens [44, 45]. Although oxycodone is not a direct serotonin reuptake inhibitor like other opioids of the phenylpiperidine class (e.g., fentanyl, dextromethorphan, etc.) [46], it has been shown in several case reports to lead to serotonin syndrome when mixed with other serotonin-releasing agents [46–49]. These reports have posited mechanisms for oxycodone-induced serotonin transmission into the nucleus accumbens, but additional mechanistic studies are needed to elucidate the true factors underlying serotonin release. Previous studies using microdialysis have demonstrated that the prototypical μ -opioid agonist morphine induces serotonin efflux into the nucleus accumbens by acting at the dorsal raphe nucleus (DRN) [50–53], likely via μ-opioid agonism of GABAergic neurons in the DRN [52]. As a morphine analog, it is possible that oxycodone induces serotonin efflux via a similar mechanism. An alternative explanation is that a metabolite of oxycodone, oxymorphone, exerts agonism of κ-opioid receptors, activation of which has been shown to reduce serotonin clearance through inhibition of serotonin transporter function in the rodent dorsal striatum and nucleus accumbens [54, 55]. This reduction of clearance would thereby increase tonic 5-HT concentrations in the nucleus accumbens.

Where previous studies have primarily failed is when attempting to resolve mixtures of DA and NE into their component concentrations, especially in vivo. These previous studies worked with phasic data. The output voltammogram of a phasic scan is a single dimensional plot of current vs. voltage. This limited information may not contain sufficient resolution to extract the small differences in redox potentials between the two monoamines. In contrast, the M-CSWV waveform consists of a series of square waveforms superimposed on a staircase sweeping potential. The advantage of this morphology is that each M-CSWV scan contains a greater number of current measurements within the redox range of DA and NE. These measurements are all compiled into a high-dimensional voltammogram, allowing for a higher-resolution analysis of the current vs. voltage behavior of the monoamines in solution. This increased resolution may allow post-processing algorithms to more accurately resolve the small differences in the voltammetric signals of the two molecules.

DiscrimNet has been shown to track expected changes in neurotransmitter levels following pharmacological intervention in vivo; however, it should be emphasized that all in vivo experiments were performed in anesthetized rodents. It is likely that in an awake, behaving organism, additional interferents to the signal would be present, including motion artifacts and other dynamic neurotransmitter changes, such as spontaneous monoamine transients, that are not present in an anesthetized animal. Further, all of our experiments were performed over the course of a single day with a fresh CFM and reference electrode for each recording; however, in a chronic implantation model, CFMs and reference electrodes would be kept within the animal for several days or weeks. Voltammogram morphology has been shown to change over such a time as a result of in vivo biofouling [56], which is also something that DiscrimNet has not been trained to handle. DiscrimNet would need additional training to handle these interferents. Such validation is the aim for future work.

DiscrimNet offers a novel method to resolve in vivo voltammetric measurements into component concentrations of DA, NE, and 5-HT in near real-time. This capability could prove to be vitally useful for in vivo study of psychiatric disease, which have been shown to arise from dysfunction in signaling of these monoamines. Further, the ability to perform real-time electrochemical measurements without the need for repetitive sampling and laboratory identification or genetic modification enables application to human surgeries, during which tonic concentrations of individual monoamines could serve as unique biomarkers to monitor intraoperatively, and possibly postoperatively using permanent electrochemical monitoring implants [1]. Indeed, deep brain stimulation devices are evolving to leverage electrophysiological biomarkers of neuromotor diseases, such as Parkinson’s Disease and essential tremor, to individualize patient treatment and stimulation parameters. An implant that would also be able to detect individual monoamine tonic concentrations would enable creation of high-dimensional patient-specific models with electrochemical and electrophysiological features of their pathophysiology, ultimately enabling higher fidelity individualized treatment.

## Data Availability

The data present within this work will be provided upon reasonable request from the authors.

## Code availability

The source code for this work will be available upon reasonable request from the authors.
